# The expression of plakoglobin is a potential prognostic biomarker for patients with surgically resected lung adenocarcinoma

**DOI:** 10.18632/oncotarget.7729

**Published:** 2016-02-25

**Authors:** Xiaobo He, Ting Zhou, Guangwei Yang, Wenfeng Fang, Zelei Li, Jianhua Zhan, Yuanyuan Zhao, Zhibin Cheng, Yan Huang, Hongyun Zhao, Li Zhang

**Affiliations:** ^1^ Department of Medical Oncology, Sun Yat-Sen University Cancer Center, Guangzhou, China; ^2^ State Key Laboratory of Oncology in South China, Guangzhou, China; ^3^ Collaborative Innovation Center for Cancer Medicine, Guangzhou, China; ^4^ Department of Oncological Radiotherapy, the Fifth Affiliated Hospital of Sun Yat-Sen University, Zhuhai, China

**Keywords:** adenocarcinoma, biomarker, immunohistochemistry, plakoglobin, prognosis in surgically resected lung adenocarcinoma

## Abstract

**Purpose:**

This study aimed to explore the relationship between plakoglobin expression and clinical data in the patients with surgically resected lung adenocarcinoma.

**Results:**

With follow-up of median 50.14 months, the average PFS and OS were 16.82 and 57.92 months, respectively. In 147 patients, recurrence or death was observed in 131 patients. According to the log-rank test, low plakoglobin expression was a significant predictor for favorable DFS (*P=0.006*) and OS (*P=0.043*). For the analyses within subgroups, high plakoglobin expression was an independent factor for reducing DFS in non-metastatic patients with resected lung adenocarcinoma (*P < 0.05*). Moreover, high plakoglobin expression was associated with poor DFS even receiving adjuvant chemotherapy (*P =0.028*) and with a shorter DFS (HR, 2.01, 95%CIs, 1.35 to 2.97, *P=0.001*) and OS (HR, 1.94, 95%CIs, 1.12 to 3.37, *P=0.019*).

**Patients and methods:**

The expression of plakoglobin in 147 primary tumor tissues was examined by using immunohistochemistry and clinical data were collected. The optimal cutoff value of immunoreactivity score (IRS) was calculated and used to divide all the patients into two groups: low-level group (IRS: 0-3, *n*=59) and high-level group (IRS: 4-12, *n*=88). Kaplan–Meier curves were applied to assess the plakoglobin expression and clinical variables. The univariate and multivariate Cox model analyses were performed to evaluate the effects of clinical factors and plakoglobin expression on disease-free survival (DFS) and overall survival (OS).

**Conclusion:**

High plakoglobin expression is an independent negative prognostic factor for patients with surgically resected lung adenocarcinoma.

## INTRODUCTION

Lung cancer is the leading common cause of cancer-related deaths around the world [1]. Approximately 80% of lung cancer cases belong to non-small-cell lung carcinoma (NSCLC) [2]. Importantly, lung adenocarcinoma is the most frequent subtype of NSCLC [3]. Curative-intent resection remains the backbone of therapeutics and increases the chance for survival in early diagnosed NSCLC [4]. Five-year survival rate ranges between 10% and 30% for patients with stage IIIA lung cancer [5]. Several randomized controlled trials reported that adjuvant chemotherapy substantially improved the survival in patients with resected NSCLC [6, 7]. However, most patients eventually experience a recurrence of cancer despite potentially curative therapy or even after complete tumor resection, so the postoperative survival rate remains unsatisfactory. In addition, for the advanced NSCLC, although targeted therapeutics have shown some promising results, these therapies are restricted to limited cases due to infrequently characterized driver mutations [8-10]. Hence, identification of prognostic and predictive factors in patients with lung adenocarcinoma is necessary for stratifying higher-risk patients for further management.

Plakoglobin (JUP, γ-catenin, gamma catenin), a member of the armadillo family proteins, is a cytoplasmic component of desmosomes [11]. It plays different cellular functions including structural roles and transcriptional activator roles [12]. Several studies have reported that plakoglobin has both positive and negative roles in various malignancies [13-15]. Previous studies seem to reveal the association between low level of plakoglobin expression and poor outcomes in NSCLC patients [16-18]. A recent study reported that γ-catenin did not predict postoperative recurrence or disease-free survival in lung cancer patients [19]. However, the relationship between the plakoglobin expression and the survival of patients with resected lung adenocarcinoma still remains controversial.

Previous study reported that circulating tumor cell (CTC) clusters are oligoclonal precursors of breast cancer metastasis and knockdown of cell junction component plakoglobin abrogates CTC cluster formation and suppresses lung metastases [20]. In the present study, we hypothesized that the plakoglobin expression in the primary tumor tissues from lung adenocarcinoma may be associated with different DFS and OS. According to the immunoreactivity score (IRS), we investigated the relationship between plakoglobin expression in patients with resected lung adenocarcinoma and their clinical outcomes.

## RESULTS

### The characteristics of NSCLC Patients

The median follow-up period was 50.14 months. The study cohort of 147 patients included 53 (36.1%) females and 88 (59.9%) non-smokers. The median age of patients was 55 years (range, 47-63). More than half of the patients (*n* = 96, 65.3%) showed pathological characteristics of moderately differentiated adenocarcinoma. Total 101 patients received postoperative chemotherapy. In addition, EGFR mutation detection was performed in 89 patients in the whole cohort. Only 45 patients were positive for EGFR mutation. The anti-EGFR drugs (gefitinib and erlotinib) were used in 70 cases. Clinicopathological characteristics of the patients are listed in Table 1.

**Table 1:** Baseline characteristics of all patients (n=147)

Characteristics	Cases (n=147)	Percentage (%)
Age (years)		
Mean	53.9	
Median	55	
Range	47 to 63	
Gender		
Female	53	36.1
Male	94	63.9
Smoking history		
Never smoking	88	59.9
Current or ever Smoking	59	40.1
Differentiation		
Well	6	4.0
Moderate	96	65.3
Poor	45	30.7
pTNM stage		
Ia	11	7.5
Ib	28	19.0
IIa	8	5.4
IIb	14	9.6
IIIa	46	31.3
IIIb	7	4.8
IV	33	22.4
pT status		
T1	25	17.0
T2	97	66.0
T3+ T4	25	17.0
pN status		
N0+ N1	79	53.7
N2+ N3	68	46.3
pM status		
M0	114	77.6
M1	33	22.4
Adjuvant chemotherapy	40	45.5
Yes	101	68.7
No	46	31.3
Serum CEA		
Normal (≤5 ng/ml)	71	48.3
Elevated (>5ng/ml)	76	51.7
EGFR mutation status		
Positive	45	30.6
Negative	44	29.9
Not performed	58	39.5
Anti-EGFR drug treatment		
Yes	70	47.6
No	77	52.4
Plakoglobin expression		
IRS 0-3	59	40.1
IRS 4-12	88	59.9

Abbreviation: pTNM, pathologic tumor–node–metastasis; IRS: immunoreactivity score; CEA, carcinoembryonic antigen. EGFR: epidermal growth factor receptor.

### Expression of plakoglobin in NSCLC and its relationship with clinicopathological variables

Cytoplasmic plakoglobin expression was visible in 111 cases (75.5%), but negative in 36 cases (24.5%) (Supplementary Table S1, Figure 2). According to the IRS cut-off value, high cytoplasmic plakoglobin expression was observed in 88 (59.9%) of the 147 patients. The relationships between clinicopathological variables and cytoplasmic plakoglobin expression are summarized in Table 2. The level of plakoglobin expression was not associated with clinicopathological variables.

**Figure 1:**
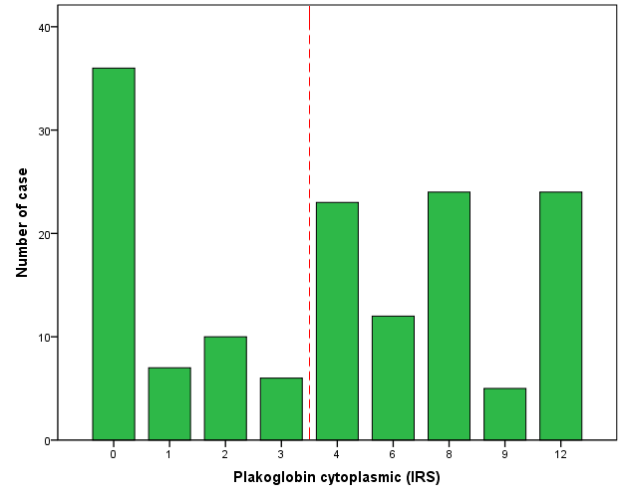
Frequency distribution of cytoplasmic plakoglobin expression The cutoff point for survival analysis is indicated as dotted lines.

**Figure 2:**
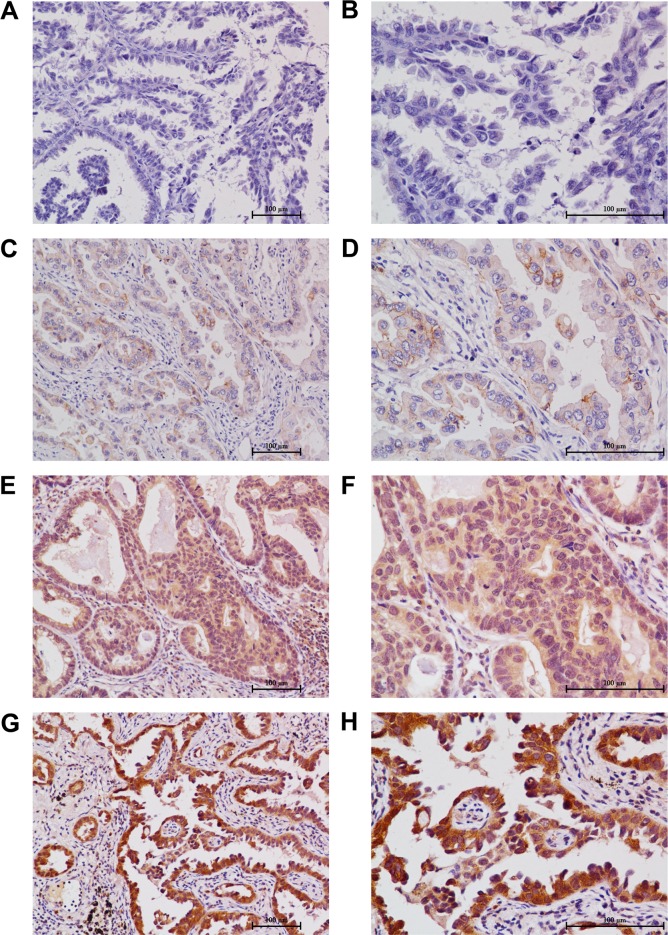
Immunohistochemical stainings of plakoglobin in the primary tumor of surgically resected lung adenocarcinoma **A.** A tumor tissue showed a negative staining of plakoglobin in all the tumor cells (200 ×). **B.** A tumor tissue showed a negative staining of plakoglobin in all the tumor cells (400 ×) (the IRS of this field: 0). **C.** the low staining of plakoglobin expression was detected in a tumor tissue (200 ×). **D.** The low staining of plakoglobin expression was detected in a tumor tissue, in which about 90% of tumor cells were observed (400 ×) (the score of this field: 4). **E.** the intermediate staining of plakoglobin expression was detected in a tumor tissue (200 ×). **F.** The intermediate staining of plakoglobin expression was detected in a tumor tissue, in which about 95% of tumor cells were observed (400 ×) (the IRS of this field: 8). **G.** the high staining of plakoglobin expression was detected in a tumor tissue (200 ×). **H.** The high staining of plakoglobin expression was detected in a tumor tissue, in which about 95% of tumor cells were observed (400 ×) (the IRS of this field: 12).

**Table 2:** Clinicopathological variables of patients according to Plakoglobin expression

Characteristics	IRS 0-3	IRS 4-12	*P* value
Age (years)			0.572^a^
Mean	54.7	53.5	
Median	55	55	
Range	47 to 62	46 to 63	
Gender			0.656^b^
Female	20 (33.9%)	33 (37.5%)	
Male	39 (66.1%)	55 (62.5%)	
Smoking history			0.913 ^b^
Never smoking	35 (59.3%)	53 (60.2%)	
Current or ever Smoking	24 (40.7%)	35 (39.8%)	
Differentiation			0.868 ^b^
Well	3 (5.1%)	3 (3.4%)	
Moderate	38 (64.4%)	58 (65.9%)	
Poor	18 (30.5%)	27 (30.7%)	
pTNM stage			0.284 ^b^
Ia	3 (5.1%)	8 (9.1%)	
Ib	12 (20.3%)	16 (18.2%)	
IIa	3 (5.1%)	5 (5.7%)	
IIb	9 (15.3%)	5 (5.7%)	
IIIa	14 (23.7%)	32 (36.4%)	
IIIb	2 (3.4%)	5 (5.7%)	
IV	16 (27.1%)	17 (19.3%)	
pT status			0.646 ^b^
T1	8 (13.6%)	17 (19.3%)	
T2	41 (69.5%)	56 (63.6%)	
T3+ T4	10 (16.9%)	15 (17.0%)	
pN status			0.439 ^b^
N0+ N1	34 (57.6%)	45 (51.1%)	
N2+ N3	25 (42.4%)	43 (48.90%)	
pM status			0.267 ^b^
M0	43 (72.9%)	71 (80.7%)	
M1	16 (27.1%)	17 (19.3%)	
Adjuvant chemotherapy			0.357 ^b^
Yes	38 (64.4%)	63 (71.6%)	
No	21 (35.6%)	25 (28.4%)	
Serum CEA			0.865 ^b^
Normal (≤5 ng/ml)	29 (49.2%)	42 (47.7%)	
Elevated (>5ng/ml)	30 (50.8%)	46 (52.3%)	
EGFR mutation status			0.329 ^b^
Positive	22 (37.3)	23 (26.1)	
Negative	15 (25.4)	29 (33.0)	
Not performed	22 (37.3)	36 (40.9)	
Anti-EGFR drug treatment			0.521 ^b^
Yes	30 (50.8)	40 (45.5)	
No	29 (49.2)	48 (54.5)	

Abbreviation: pTNM, pathologic tumor–node–metastasis; IRS: immunoreactivity score; CEA, carcinoembryonic antigen; EGFR: epidermal growth factor receptor.

a Kraskal-Wallis test

b Chi-square test

### The correlations between cytoplasmic plakoglobin expression and DFS/OS in NSCLC patients

**Figure 3:**
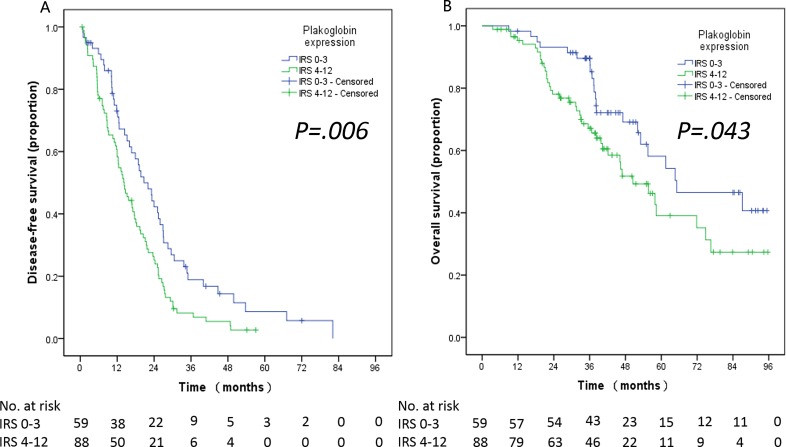
Kaplan-Meier analyses for disease-free survival (DFS) **A.** and overall survival (OS) **B.** according to the cut-off value of immunoreactivity score (IRS).

Among the 147 patients with lung adenocarcinoma, the median DFS and OS time was 16.82 and 57.92 months, respectively. A significant negative correlation was observed between plakoglobin expression and DFS (low *vs.* high plakoglobin, 20.7 *vs.* 14.30 months, *P = 0.006)* and OS (low *vs.* high plakoglobin, 65.22 *vs.* 50.46 months, *P = 0.043)* (Figure 3).

**Figure 4:**
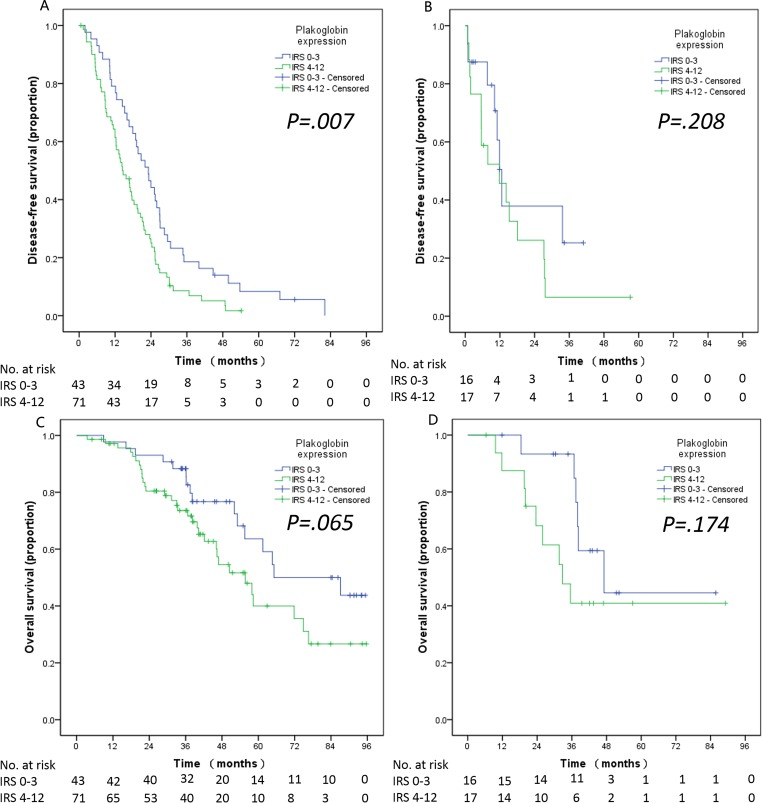
Cytoplasmic plakoglobin expression, DFS and OS are shown by different columns, the subgroups are shown in the different rows according to whether the presence of metastasis in patients before surgery or not The relationship between plakoglobin expression and DFS **A**. and OS **C**. in the patients without metastasis before surgery. The relationship between plakoglobin expression and DFS **B**. and OS **D**. and in the patients with metastasis before surgery.

**Figure 5:**
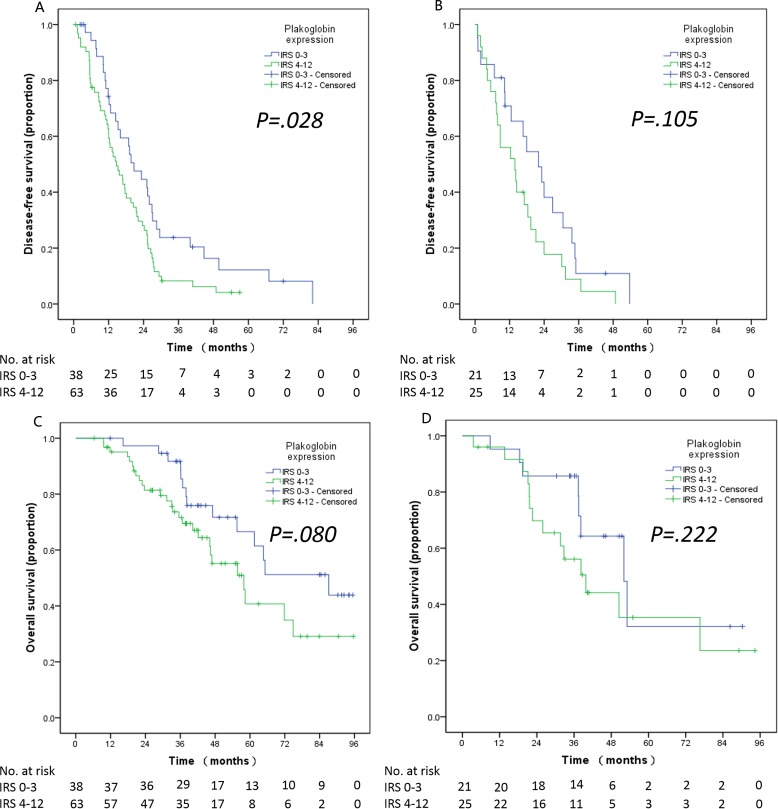
The relationship between plakoglobin expression and DFS A. and OS C. in the patients received adjuvant chemotherapy after surgery The relationship between plakoglobin expression and DFS **B**. and OS **D**. in the patients without adjuvant chemotherapy after surgery.

In the analysis of the subgroups, firstly, stratified analyses according to the metastasis classification (M-stage) revealed that the significant effect was only observed in the patients with non-metastasis (M0). Patients with non-metastasis (M0) had a shorter DFS when cytoplasmic plakoglobin expression was high (low *vs.* high plakoglobin, DFS, 23.16 *vs.* 14.68 months, *P = 0.007*, Figure 4A). Meanwhile, the survival curve of OS revealed that high cytoplasmic plakoglobin expression was associated with worse outcomes in patients with non-metastasis (M0) (low *vs.* high plakoglobin, OS, 87.13 *vs.* 55.69 months, *P = 0.065*, Figure 4C). There was no significant correlation between the cytoplasmic plakoglobin expression (low *vs.* high plakoglobin) and the clinical outcomes in patients with metastasis (M1), (DFS, 12.65 *vs.* 11.76 months, *P = 0.208*, Figure 4B; OS, 47.15 *vs.* 32.72 months, *P = 0.174*, Figure 4D). Secondly, based on whether received adjuvant chemotherapy or not, we found that high plakoglobin expression was significantly associated with shorter PFS in the patients who received adjuvant chemotherapy (low *vs.* high plakoglobin, DFS, 20.69 *vs.* 14.69 months; *P = 0.028*, Figure 5A), and this effect was obvious but not signficant in the patients who didn't receive adjuvant chemotherapy (low *vs.* high plakoglobin, DFS, 22.05 *vs.* 13.80 months; *P = 0.105*, Figure 5B). For OS, we also observed similar correlation between low plakoglobin expression and better OS in the patients whoever received adjuvant chemotherapy (low *vs.* high plakoglobin, OS, 87.13 *vs.* 57.92 months, *P = 0.080*, Figure 5C) or didn't receive adjuvant chemotherapy (low *vs.* high plakoglobin, OS, 52.07 *vs.* 39.82 months, *P = 0.222*, Figure 5D).

The univariate analysis demonstrated that high plakoglobin expression was significantly correlated with reduced DFS (Hazard ratio [HR], 1.65, 95%CIs, 1.15 to 2.37, *P = 0.007*) and OS (HR, 1.70, 95%CIs, 1.01 to 2.86, *P = 0.046*). Additionally, the abnormal elevated carcinoembryonic antigen (CEA) was associated with poor DFS (HR, 1.44, 95%CIs, 1.02 to 2.03, *P = 0.039*), but not OS (*P = 0.204*). The TNM-stage also showed a significant impact on all the patients' OS in univariate analysis (*P = 0.038*). Clinicopathological parameters such as age, gender, smoking status, differentiation and adjuvant chemotherapy were not of independent prognostic values (Table 3). Moreover, all the potential dangerous factors were analyzed by the multivariate Cox proportional hazards model, we found that the cytoplasmic plakoglobin expression was an independent predictor for DFS (HR, 2.01, 95%CIs, 1.35 to 2.97, *P = 0.001*) and OS (HR, 1.94, 95%CIs, 1.12 to 3.37, *P = 0.019*). Additionally, adjuvant chemotherapy (*P = 0.027*) and TNM-stage (*P = 0.005*) were also an independent predictor for OS (Table 4).

**Table 3:** Univariate and multivariate analyses for variables considered for disease-free survival (Cox proportional hazard regression model)

	DFS Univariate analysis			DFS Multivariate analysis		
Characteristics	95.0% CIs	HR	*P* value	95.0% CIs	HR	*P* value
Age (years)						
<55		1.0 (ref.)			1.0 (ref.)	
≥55	0.77 to 1.54	1.09	0.630 ^a^	0.63 to 1.33	0.91	0.631 ^b^
Gender						
Female		1.0 (ref.)			1.0 (ref.)	
Male	0.99 to 1.02	1.38	0.084 ^a^	0.94 to 2.40	1.50	0.090 ^b^
Smoking history						
Never smoking		1.0 (ref.)			1.0 (ref.)	
Current or ever Smoking	0.83 to 1.67	1.18	0.359 ^a^	0.60 to 1.44	0.93	0.755 ^b^
Differentiation						
Well		1.0 (ref.)			1.0 (ref.)	
Moderate	0.41 to 2.20	0.95	0.909 ^a^	0.33 to 1.94	0.80	0.831 ^b^
Poor	0.55 to 3.07	1.30	0.549 ^a^	0.47 to 2.91	1.17	0.743 ^b^
pTNM stage, continuous	0.96 to 1.16	1.06	0.250 ^a^	0.95 to 1.16	1.05	0.343 ^b^
pT status						
T1		1.0 (ref.)		-	-	-
T2	0.62 to 1.53	0.97	0.909 ^a^	-	-	-
T3+ T4	0.55 to 1.81	1.00	0.998 ^a^	-	-	-
pN status						
N0+ N1		1.0 (ref.)		-	-	-
N2+ N3	1.00 to 2.00	1.41	0.051 ^a^	-	-	-
pM status						
M0		1.0 (ref.)		-	-	-
M1	0.74 to 1.83	1.16	0.512 ^a^	-	-	-
Adjuvant chemotherapy						
Yes		1.0 (ref.)			1.0 (ref.)	
No	0.78 to 1.63	1.12	0.532 ^a^	0.88 to 2.03	1.33	0.177 ^b^
EGFR mutation status						
Positive		1.0 (ref.)				
Negative	0.56 to 1.37	0.88	0.566 ^a^	0.42 to 1.22	0.71	0.213 ^b^
Not performed	0.63 to 1.43	0.95	0.802 ^a^	0.42 to 1.06	0.67	0.087 ^b^
Anti-EGFR drug treatment						
Yes		1.0 (ref.)			1.0 (ref.)	
No	0.60 to 1.19	0.84	0.327 ^a^	0.06 to 1.44	0.98	0.899 ^b^
Serum CEA						
Normal (≤5 ng/ml)		1.0 (ref.)			1.0 (ref.)	
Elevated (>5ng/ml)	1.02 to 2.03	1.44	0.039 ^a^	0.95 to 2.02	1.38	0.092 ^b^
Plakoglobin expression						
IRS 0-3		1.0 (ref.)			1.0 (ref.)	
IRS 4-12	1.15 to 2.37	1.65	0.007 ^a^	1.35 to 2.97	2.01	0.001 ^b^

Abbreviation: pTNM, pathologic tumor–node–metastasis; IRS: immunoreactivity score; EGFR: epidermal growth factor receptor; CEA, carcinoembryonic antigen; HR, hazard ratio; CIs, confidence intervals.

a Univariate Cox proportional hazard regression

b Multivariate Cox proportional hazard regression

**Table 4:** Univariate and multivariate analyses for variables considered for overall survival (Cox proportional hazard regression model)

	OS Univariate analysis			OS Multivariate analysis		
Characteristics	95.0% CIs	HR	*P* value	95.0% CIs	HR	*P* value
Age (years)						
<55		1.0 (ref.)			1.0 (ref.)	
≥55	0.99 to 2.69	1.63	0.056 ^a^	0.97 to 2.90	1.68	0.063 ^b^
Gender						
Female		1.0 (ref.)			1.0 (ref.)	
Male	0.86 to 2.62	1.50	0.152 ^a^	0.64 to 2.72	1.32	0.461 ^b^
Smoking history						
Never smoking		1.0 (ref.)			1.0 (ref.)	
Current or ever Smoking	0.85 to 2.28	1.39	0.194 ^a^	0.62 to 2.29	1.19	0.599 ^b^
Differentiation						
Well		1.0 (ref.)			1.0 (ref.)	
Moderate	0.52 to 9.04	2.17	0.289 ^a^	0.34 to 6.66	1.50	0.598 ^b^
Poor	0.89 to 16.17	3.80	0.071 ^a^	0.54 to 11.32	2.46	0.247 ^b^
pTNM stage, continuous	1.01 to 1.32	1.15	0.038 ^a^	1.06 to 1.41	1.22	0.005 ^b^
pT status						
T1		1.0 (ref.)		-	-	-
T2	0.56 to 2.39	1.16	0.691 ^a^	-	-	-
T3+ T4	0.83 to 4.43	1.92	0.129 ^a^	-	-	-
pN status						
N0+ N1		1.0 (ref.)		-	-	-
N2+ N3	0.60 to 1.63	0.99	0.980 ^a^	-	-	-
pM status						
M0		1.0 (ref.)		-	-	-
M1	0.83 to 2.68	1.49	0.184 ^a^	-	-	-
Adjuvant chemotherapy						
Yes		1.0 (ref.)			1.0 (ref.)	
No	0.89 to 2.52	1.50	0.129 ^a^	1.08 to 3.48	1.94	0.027 ^b^
EGFR mutation status						
Positive		1.0 (ref.)			1.0 (ref.)	
Negative	0.39 to 1.57	0.79	0.494 ^a^	0.33 to 1.64	0.73	0.452 ^b^
Not performed	0.63 to 2.00	1.12	0.695 ^a^	0.37 to 1.42	0.73	0.350 ^b^
Anti-EGFR drug treatment						
Yes		1.0 (ref.)			1.0 (ref.)	
No	0.44 to 1.20	0.73	0.214 ^a^	0.46 to 1.41	0.81	0.450 ^b^
Serum CEA						
Normal (≤5 ng/ml)		1.0 (ref.)			1.0 (ref.)	
Elevated (>5ng/ml)	0.84 to 2.27	1.38	0.204 ^a^	0.63 to 1.88	1.09	0.771 ^b^
Plakoglobin expression						
IRS 0-3		1.0 (ref.)			1.0 (ref.)	
IRS 4-12	1.01 to 2.86	1.70	0.046 ^a^	1.12 to 3.37	1.94	0.019 ^b^

Abbreviation: pTNM, pathologic tumor–node–metastasis; IRS: immunoreactivity score; CEA: carcinoembryonic antigen; EGFR: epidermal growth factor receptor; HR: hazard ratio; CIs: confidence intervals.

a Univariate Cox proportional hazard regression

b Multivariate Cox proportional hazard regression

## DISCUSSION

In this study, we demonstrated that high cytoplasmic plakoglobin expression in tumor tissues is significantly associated with poor DFS and OS in the patients with resected lung adenocarcinoma. To our best knowledge, it is controversial whether plakoglobin is an oncogene or tumor suppressor in some *in vitro* experiments. Several studies indicate that plakoglobin has oncogenic activities. Overexpression of plakoglobin in RK3E promotes neoplastic transformation because plakoglobin overexpression induces the upregulation of c-Myc and activation of Tcf/Lef signaling [14]. Similarly, the DSG3-plakoglobin-TCF/LEF-Myc/cyclin D1/MMP signaling pathway facilitates cancer growth and invasion [21]. In addition, plakoglobin has also been regarded as a tumor suppressor, inhibiting tumor growth, migration, and invasion [22, 23]. Sechler et al. reported the anti-migratory effects of γ-catenin are driven by the expression of hepatocyte growth factor activator inhibitor Type I (HAI-1 or SPINT-1), an upstream inhibitor of the c-MET signaling pathway in NSCLC cell lines [23]. Thus, previous evidence reveals that plakoglobin functions as a two-edge sword: oncogene or tumor suppressor, which depends on the cellular context and the activated downstream signaling pathways [24]. However, the prognostic significance of plakoglobin in patients with resected lung adenocarcinoma remains to be defined.

Previous literature reported that deficient expression of plakoglobin appears to be an important event in the progression of NSCLC [16-18], which is not consistent with our findings. However, the discrepancy can be explained for the following reasons: (1) here we collected 147 tumors merely from lung adenocarcinoma; (2) the IHC staining method was used to analyze plakoglobin expression in tumor tissues. IRS of plakoglobin expression was determined by in the combination of the percentage and intensity of positively stained cells. All patients were divided into two groups according to the optimal cutoff value. (3) High plakoglobin expression in primary tumor may form the circulating tumor cell clusters that contribute to invasion and metastases in breast cancer and colorectal cancer [20, 25]. (4) In this study, we observed the expression of plakoglobin in the membrane and cytoplasm. As the component of intercellular adhesive junctions, some studies revealed that the membrane and cytoplasm plakoglobins are the components of the cytoplasmic plaque of desmosomes [26-28]. Furthermore, the deposit product of the immunohistochemical substrate might not be always exactly localized at the site of antibody binding, but it would variably diffuse into the vicinity of the binding site. Therefore, it is hard to exclude that the observed transmutation in the subcellular localization of the staining results from the surgical specimen. According to our results, the intensity and the area of plakoglobin-positive tumor cells were very close. So we used the cytoplasmic plakoglobin expression as a parameter to figure out clinical relevance. This evidence implicates that high plakoglobin expression status in primary tumor can predict invasive status and risk of recurrence in lung adenocarcinoma patients.

Based on the analysis in subgroups, we found that the high plakoglobin expression was associated with shorter DFS and OS in patients without metastasis, but this correlation was not observed in the patients with metastasis due to limited number of patients (n = 31). Pantel *et al*. reported that reduced plakoglobin expression might be an important event in the progression of NSCLC [16]. However, they only included 44 patients with lung adenocarcinoma and did not analyze the relationship between plakoglobin expression and DFS. Additionally, their IHC results only considered the percentage of positive tumor cells. Another study reported that γ-catenin expression was a positive prognostic factor by using univariate survival analysis but not multivariate analysis [17]. The discrepancy between this study and previous finding may be caused by different follow-up period in all patients. Two studies explored the molecular mechanisms that γ-catenin inhibits the proliferation of lung cancer cells *in vitro* [23, 29]. However, these results only support the suppressive role of γ-catenin in single tumor cells, but not further clarify the effect of γ-catenin on the formation of CTC clusters. In other words, the correlation between high plakoglobin expression and worse survival of lung adenocarcinoma may be not related to oncogenic or tumor-suppressive function of plakoglobin. Instead, being an adhesion molecule, high plakoglobin expression enables tumor cells to aggregate together and move in clusters in the bloodstream, which increases the chance of metastasis and results in worse survival of lung adenocarcinoma. It's probably helpful to detect the CTC clusters in the blood of patients with lung adenocarcinoma, which can further illustrate the role of plakoglobin. Our results reveal that different plakoglobin expression in primary tumors may influence the outcome of patients with lung adenocarcinoma.

Several clinical implications can be predicted from our findings. (1) IHC method was used in this study to obtain the cutoff value of plakoglobin expression in lung adenocarcinoma and provided the basis for future screening of patients in clinical trials. (2) To our best knowledge, it seems that no single marker can independently predict tumor prognosis, here we found that the combination of plakoglobin expression and several markers may provide a powerful method for predicting patients' outcomes. (3) Our results indicate that lowerplakoglobin expression in primary tumor is related to longer DFS and OS in the patients with lung adenocarcinoma. (4) Based on the analyses of subgroups, adjuvant chemotherapy plus the anti-plakoglobin treatment may be a better strategy for patients with resected lung adenocarcinoma but without obvious metastasis, which may provide strong evidence for anti-plakoglobin therapy in future clinical trials in the patients with lung adenocarcinoma. However, this study has a few limitations: (1) Our results need to be verified *in vitro* and *in vivo* studies; (2) This retrospective study was carried out at a single institution. These findings should be interpreted cautiously and need to be confirmed in large prospective trials

In conclusion, high plakoglobin expression in primary tumors of lung adenocarcinoma is associated with shorter DFS and OS, suggesting that plakoglobin is a potential biomarker for the prognosis of lung adenocarcinoma and plakoglobin may be considered as a potential therapeutic target in the patients with lung adenocarcinoma.

## PATIENTS AND METHODS

### Patients and treatments

The retrospective trial was performed in 147 histologically diagnosed NSCLC patients who underwent surgical resection of primary tumor betweeen October 2007 to September 2012, at Sun Yat-Sen University Cancer Center. All enrolled patients were diagnosed as lung adenocarcinoma according to the classification of World Health Organization [30]. Pathological Tumor-Node-Metastasis classification (TNM) and staging were determined by the international staging system [31]. The assessments of pretreatment were performed based on complete medical history and a series of examinations such as complete blood cell count, serum biochemistry, chest X-ray, computed tomography (CT) scans of the chest and abdomen. The postoperative adjuvant chemotherapy using cisplatin-based regimens was performed in the patients who could accept and tolerate treatment. The EGFR mutation detection has been performed as an examination for the NSCLC patients since December of 2008 in our hospital and the results of EGFR mutation detection were recorded in hospital information system. The study was approved by the Medical Ethics Committee of the Cancer Center at Sun Yat-Sen University.

### Follow-up

After the completion of primary treatments, patients were followed up every 3-6 months during the first 2 years and every 6-12 months thereafter. The radiological examination was conducted at all time points. The survival status was obtained at clinical visits and by direct phone call to the patients or their family.

### Immunohistochemistry

The formalin-fixed paraffin sections at 4 μm thickness were dewaxed in xylene and rehydrated through graded alcohol. For epitope retrieval, the sections were heated in EDTA solution (pH = 8) for 10 min. Endogenous peroxidase activity was blocked by the treatment of 0.3% hydrogen peroxide for 15 min. The sections were preincubated with blocking solution containing normal goat serum for 30 min, then incubated with anti-plakoglobin antibody (Abcam, ab119908, Cambridge, MA, 100 dilution) overnight at 4°C in a moist chamber. After thrice washes, the sections were incubated with biotinylated secondary antibody (Dako REAL EnVision™/HRP, Rabbit/Mouse (ENV) K5007, DakoCytomation, Glostrup, Denmark) for 30 min at 37°C. The color was developed by the incubation with the mixture of Dako Real™ Substrate Buffer and Dako Real™ DAB+ Chromogen. All sections were counterstained with Mayer's hematoxylin. As a negative control, the primary antibody was replaced by normal rabbit serum.

Cytoplasmic plakoglobin expression was evaluated by two independent pathologists (Drs. Xiaobo He and Zelei Li) who were blind to the clinical data of all patients. The scoring of cytoplasmic plakoglobin expression was conducted according to the following criteria: (a) the percentage of positive tumor cells (score 0, 0%; 1, score 1, 1%-25%; score 2, 26%-50%; score 3, 51%-75%; score 4, > 75%), (b) staining intensity (score 0, negative staining; score 1, low staining: light yellow; score 2, intermediate staining: yellow brown; and score 3, high staining: brown). Cytoplasmic plakoglobin expression index was calculated by a × b, with the score range from 0 to 12 (Figure 1). Five separate fields (400×) were assessed. If a disagreement occurred in some samples, the slides were reviewed again and a consensus was reached. To appropriately predict DFS, the optimal cutoff point for cytoplasmic plakoglobin expression (high *vs*. low) was determined by an automatic method (Cutoff Finder: http://molpath.charite.de/cutoff/assign.jsp) [32]. The definition for high cytoplasmic plakoglobin expression was immunoreactivity score (IRS) ≥ 4.

### Statistical analysis

Median follow-up was estimated by the reverse Kaplan-Meier method [33]. The first end point of this analysis was disease-free survival (DFS), defined as the time from diagnosis until recurrence of tumor (first local, regional, distant recurrence) or death from any cause, whichever came first. The second end point was overall survival (OS), defined as the time from diagnosis to the date of death from any cause.

The relationships between the cytoplasmic plakoglobin expression and clinical characteristics were evaluated by using χ2 tests. DFS, OS and the 95% confidence intervals (CIs) among groups and subgroups were estimated by the Kaplan-Meier method [34]. The Cox proportional hazards models for univariate and multivariate analyses were used to evaluate the prognostic role of cytoplasmic plakoglobin expression in DFS and OS [35]. Because the number of patients in some subgroups of different stages was relatively small, hazard ratio (HR) was estimated to assess the discriminatory ability of the pTNM stage groups. Statistical analyses were performed using Empower (R) (www.empowerstats.com, X&Y solutions, Inc., Boston, MA), R-project (http://www.R-project.org) and Statistical Package for Social Sciences (SPSS) 21.0 software (IBM, Armonk, NY). The level of statistical significance was set to 0.05.

## SUPPLEMENTARY MATERIAL TABLE


