# Supplementary material for: The expression of plakoglobin is a potential prognostic biomarker for patients with surgically resected lung adenocarcinoma
Source: Oncotarget. 2016 Feb 25;7(12):15274–87. doi: 10.18632/oncotarget.7729 (PMC4924786; doi:10.18632/oncotarget.7729)
Supplement: Supplementary file 1 [file oncotarget-07-15274-s001.pdf]

The expression of plakoglobin is a potential prognostic biomarker for patients with surgically resected lung adenocarcinoma

Supplementary Material

Supplementary Table 1: The distribution of immunoreactivity score (IRS) for plakoglobin expression in all patients.

immunoreactivity score	Case	Percentage
0	36	24.5
1	7	4.8
2	10	6.8
3	6	4.1
4	23	15.6
6	12	8.2
8	24	16.3
9	5	3.4
12	24	16.3
